# Bacterial biosynthetic gene clusters

**DOI:** 10.1042/EBC20260074

**Published:** 2026-08-19

**Authors:** Francisco Barona-Gómez

**Affiliations:** Evolution of Microbial Chemodiversity Laboratory, Institute of Biology, Leiden University, Sylviusweg 72, 2333 BE Leiden, The Netherlands

**Keywords:** Biosynthetic Gene Cluster, Natural Product Biosynthesis, Secondary Metabolism, Specialised Metabolite

## Abstract

The concept of biosynthetic gene clusters (BGC) has been at the centre of the study of bacterial secondary metabolism during this century, allowing a major transition in how this elusive complex trait was historically investigated, from individual cases to currently focusing on general emerging principles. During the last century, marked by a community of practitioners consisting of chemists and microbiologists mainly using analytical chemistry and microbiological techniques, the field has evolved into a richer and more diverse scientific community using genetic, genomic, chemical, and structural biology and evolutionary and ecological approaches, with broader implications. This transition coincides not only with the emergence and consolidation of the so-called ‘omics’ sciences but also with the terms ’natural products’, mainly used by biological chemists exploring biotechnological applications of secondary metabolites, and ‘specialised metabolites’, embraced by microbiologists aware of the broader ecological and evolutionary dimensions of the metabolites encoded by BGCs.

In the present special issue, the concept of bacterial biosynthetic gene clusters (BGC) is revisited by secondary metabolite scientists that have made these metabolites their primary research topic. A total of 16 essays contributed by scientists from North and South America, Europe, Asia, and Oceania review large bodies of literature and provide novel views of discovery tools [1–4], genetic and chemical regulation [5–8], function and ecology [9–12], and evolution and chemical diversification [13–16] of the metabolites encoded by BGCs. While the presentation order of these essays of the present special issue reflects a reasonable classification, in many cases, these manuscripts cross talk and overlap in the disciplines, approaches, and concepts they cover. This reflects the centrality of BGCs to understand bacterial secondary metabolism and how, in a more rational fashion, we can continue to benefit from the metabolic services bacteria and their biosynthetic pathways can offer us to address the many challenges we face, from tackling pandemics to guaranteeing food security.

Since the ‘golden age’ of antibiotic discovery in the middle of the last century, the field has heavily depended on, and still does, on screening the bioactivity of secondary metabolites. This is despite the advent of the so-called genome mining of natural products revolution, which has revealed an overwhelming diversity and number of BGCs to be characterised. As this computational approach has been addressed by many recent and comprehensive reviews (and special journal issues) this topic is not covered here. Back to screening, to overcome the many problems and challenges related to approaches based on bioactivity, and by embracing the architecture of BGCs, Licona-Cassani and co-workers provide an overview of the use of biosensors to uncover known and unknown chemical diversity [1]. To further expand the known BGC diversity and assign potential gene function, Pidot and co-workers elaborate on the use of transposon mutagenesis of model and environmental strains [2]. In back-to-back contributions, D'Agostino and co-workers provide detailed approaches to the heterologous expression and analysis of multidomain and modular enzymes (NRPS and PKS) and their complete pathways, as encoded by BGCs [3,4]. These four contributions provide the frontline of BGC discovery based on genetic tools, only possible due to our understanding of BGC structure and the revolution brought about by genome mining approaches.

Since the first BGCs were cloned and linked with their metabolite products, the complexity and intricate logic of these loci encoding non-linear pathways as well as extra functional genes mediating their regulation, transport, and resistance, caught scientists' attention. The regulation of BGCs, a complex trait that responds to environmental and developmental cues, is highlighted by the concept of bacterial biosynthetic superclusters, an emerging concept that is reviewed here, for the first time, by Camara and co-workers [5]. As collectives of BGCs, superclusters may encode for functionally complementary metabolites that demand hierarchical and integrated regulatory regimens. This can involve chemically driven genetic regulation, as highlighted by lactone-signalling systems reviewed by Arakawa and co-workers [6]. The genes encoding for the biosynthetic enzymes and receptor genes of these signalling metabolites can be found within BGCs and superclusters. Transcriptional regulation of BGCs has been thoroughly investigated, but it is not until recently that unprecedented translational mechanisms as a hub for secondary metabolism is starting to emerge, as addressed by Straight and co-workers [7]. Further expanding the regulatory possibilities of BGCs, their expression has been unambiguously linked with alternative cell types, cell densities, and compartments within the bacterial colony, as highlighted by the contribution of Elliot and co-workers [8]. The diversity of regulatory mechanisms characterised to date, including many that remain to be fully understood, speaks to the ecological complexity of BGCs and their metabolite products.

The later point about the ecological complexity of BGCs is elegantly addressed by the contributions by Rigali [9], Ueda [10], Chevrette and co-workers [11], and Kovács [12]. While Rigali asks the questions of when, where, and why specialised metabolites are produced, and how function can be linked to expression profiles of BGCs, Ueda provides an example of a metabolite with multiple functions yet encoded by the same BGC. Arguably, the ecological function(s) of secondary metabolism has received the largest interest in recent years, providing a biologically meaningful context to BGCs. Indeed, after careful analysis of the experimental evidence derived from synthetic bacterial communities isolated from diverse environmental settings, the contributions by the Chevrette group and Kovács converge to an emerging view about how BGCs provide the foundations for bacterial interactions to go beyond negative interactions as expected from antibiotics. Even in oversimplified microbial ecology experimental systems, unexpected responses involving differential BGC activation and positive interactions, as well as biotransformations outside the bacterial colony, have been documented and discussed by these authors. This includes interkingdom cases even when the present special issue focuses only on bacterial and not fungal BGCs.

Since having the honour of co-chairing the International Symposium on the Biology of Actinomycetes in 2011, under the general theme of the Evolution of Actinomycetes [17], evolutionary thinking has been more prominent in the field of bacterial secondary metabolism beyond pure speculation. Natural product evolution through horizontal gene transfer (HGT) is now well acknowledged but also properly dimensioned in the context of vertical evolution. In this context, several genetic mechanisms acting as drivers of the chemical diversification encoded by BGCs are discussed by Zotchev [13]. The possibility of HGT is further embraced by the contribution by Hoskisson and co-workers [14], who discuss the appearance and maintenance of multiple BGCs in the context of internal adaptive conflicts and kin selection. In their essay, these authors propose adoption of the so-called ‘greenbeard’ evolutionary model driven by HGT events. The chemical diversification encoded by BGCs is further explored by means of the evolutionary fitness landscape by Nivina and Thiel Pizarro [15] with an emphasis on the genetic mechanisms driving BGC genetic diversity. Similarly, with an emphasis on the finest level of chemical diversification, the final review identifies a knowledge gap in the sense that the one-strain many compounds framework and enzyme promiscuity, alone, cannot account for the chemical diversity revealed by state-of-the-art metabolomics and feature-based molecular networking. In our essay [16], we postulate a plausible genetically encoded mechanism involving the stochastic activity of enzymes, in agreement with the Dynamic Chemical Matrix evolutionary hypothesis and a new paradigm of multiple BGCs—a plethora of metabolites.

I hope readers find this collection of essays useful as an entry point to the study of BGCs, but also to help identify emerging principles and opportunities while reviewing a vast body of literature related to the discovery, regulation, ecology, and evolution of their cognate metabolite products. As a cohesive genetic element, BGCs have provided many opportunities to develop approaches for their discovery and characterisation. There is no doubt that BGCs provide a blueprint of the biology of the metabolites they encode for that has been translated into impactful biotechnologies. What would have been the current state of bacterial secondary metabolism as a field without BGCs is hard to imagine, but most likely a less successful one. This question, more than a rhetoric one, provides an interesting reflection point of what may lie ahead: how do BGCs, and the chemical diversity they encode, cross-talk at the cellular, colony, and community levels? How many pathways not encoded by BGCs remain to be discovered? What can be learned from the study of BGCs to approach unassociated biosynthetic gene (UBG) pathways that we know exist but have been neglected? What new paradigms lie ahead with regard to the genetics, chemistry, ecology, and evolution of the metabolite products produced by genetically dispersed UBG pathways? However hard these questions may seem, the study and lessons provided by more than two decades of intensive research into bacterial BGCs provide a strong foundation to embark on these and other challenges of the so-called bacterial ‘secondary’ metabolism.

## Open Access

This article has been published open access under our Subscribe to Open programme, made possible through the support of our subscribing institutions, learn more here: https://portlandpress.com/pages/open_access_options_and_prices#conditional.

## Abbreviations

BGC: biosynthetic gene cluster
HGT: horizontal gene transfer
UBG: unassociated biosynthetic gene
